# Supplementary material for: Dry synthesis of bi-layer nanoporous metal films as plasmonic metamaterial
Source: Nanophotonics. 2024 Mar 12;13(7):1159–67. doi: 10.1515/nanoph-2023-0942 (PMC11501558; doi:10.1515/nanoph-2023-0942)
Supplement: Supplementary file 1 — Supplementary Material Details [file j_nanoph-2023-0942_suppl_001.docx]

**SUPPORTING INFORMATION**

**Dry synthesis of bi-layer nanoporous metal films as plasmonic metamaterial**

Vincenzo Caligiuri, Hyunah Kwon, Andrea Griesi, Yurii P. Ivanov, Andrea Schirato, Alessandro Alabastri, Massimo Cuscunà, Gianluca Balestra, Antonio De Luca, Tlek Tapani, Haifeng Lin, Nicolò Maccaferri, Roman Krahne, Giorgio Divitini, Peer Fischer and Denis Garoli *

**Supporting note #1; Additional Ellipsometries**

The dielectric permittivity of the bare Ag and bare Au nanoporous layers is provided here, to confirm that the single-metal systems manifest a significantly different optical behavior than the bi-metallic ones. The imaginary part of the dielectric permittivity of the nanoporous Ag layer, retrieved by modeling the experimentally measured ellipsometric angles, has been modeled as a convolution of Gaussian oscillators whose expression for the imaginary part is provided in Eq.1 and 2 of the main manuscript. The parameters for each oscillator are reported in **Table S1:**

**Table 1.** Fitting parameters for the Gaussian oscillators used to fit the dielectric permittivity of the nano-porous Ag samples.

|  | ***E_0_* (eV)** | ***γ_i_* (eV)** | ***A_i_*** |
| --- | --- | --- | --- |
| **Oscillator 1** | 2.223 | 1.858 | 15.800 |
| **Oscillator 2** | 0.837 | 1.316 | 10.600 |
| **Oscillator 3** | 3.329 | 0.265 | 1.436 |
| **Oscillator 4** | 3.815 | 0.167 | 7.897 |
| **Oscillator 5** | 3.996 | 0.763 | 10.545 |

The associated real part has been retrieved via Kramers-Kronig relations. The obtained results are shown in **Figure S1a** where the real part of the dielectric permittivity is shown as a solid black curve while the imaginary part as a red curve. The ellipsometrical angles Ψ and Δ together with their fit are shown in **Figure S1b,c**.


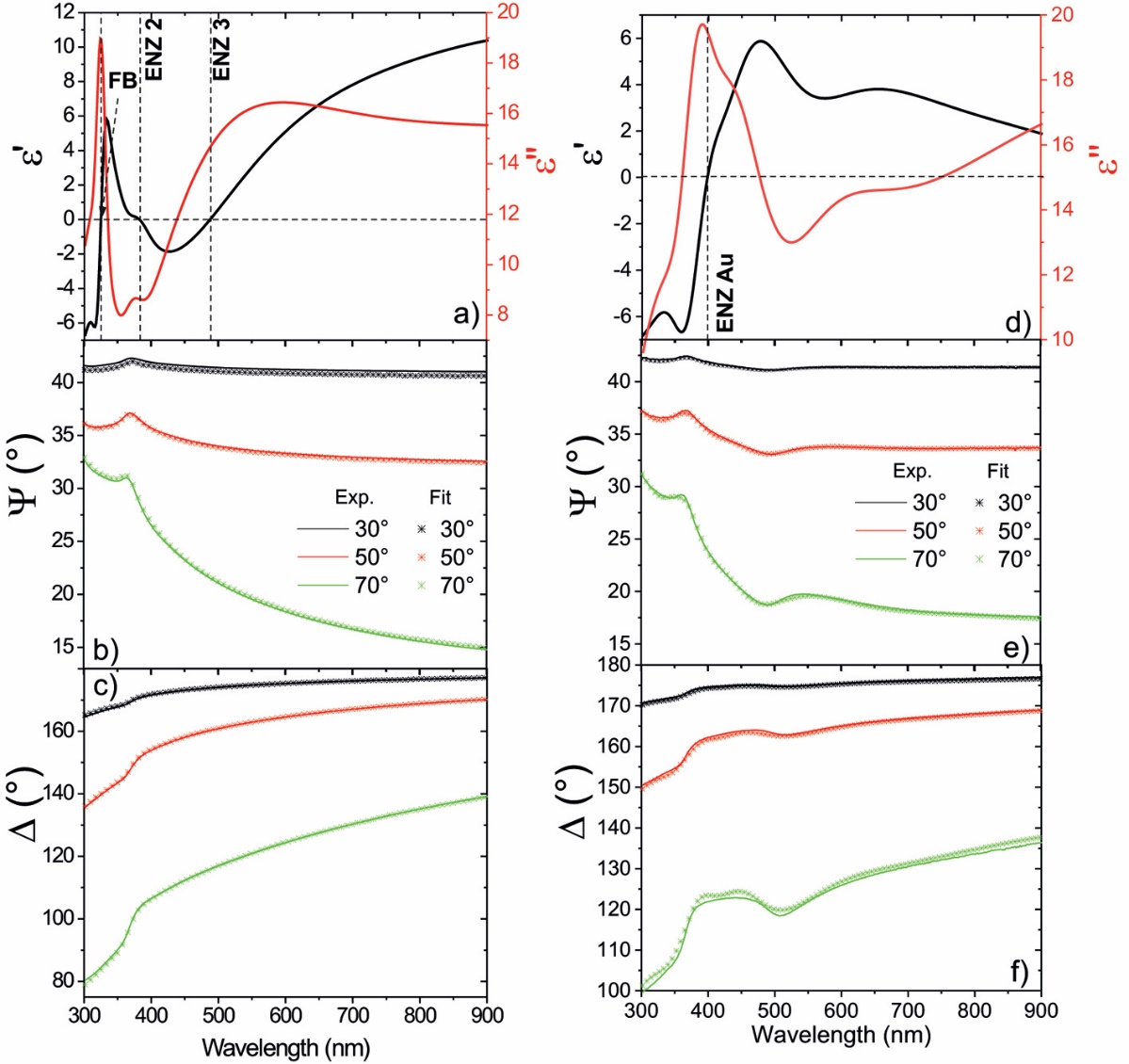


**Figure S1**: real (red curves) and imaginary (back curves) of the dielectric permittivity of (a) Ag and (d) Au nano-porous layers together with the associated experimental (solid curves) and fitted (asterisks) ellipsoemtrical angles ψ (b,e) and Δ (c,f)

The obtained dielectric permittivity significantly differs from that of pure Ag. As a signature of the presence of Ag, the effective permittivity of the nano-porous Ag layer manifests an Epsilon Near Zero transition at 327 nm (labeled as “FB” in **Figure S1a**), corresponding to the well-known Ferrel-Berreman (FB) mode.^1,2^ Noticeably, for the pure Ag layer the FB mode individuates an epsilon-near-zero (ENZ) transition from a dielectric behavior (ε’ > 0) to a metallic (ε’ < 0) one. For the nano-porous Ag layer the situation is reversed. This ENZ transition determines a switching from a metallic to a dielectric behavior. Two additional ENZ transitions are present around 380 nm (labeled “ENZ 2” in **Figure S1a**) and 490 nm (labeled “ENZ 3” in **Figure S1a**), between which a high-lossy metallic band is found (**Figure S1a**). Above the ENZ transition at 490 nm, the layer behaves as a highly-lossy dielectric with a large imaginary dielectric permittivity. For the case of nano-porous Au, a similar analysis has been carried out, revealing once more substantially different dielectric permittivity than that of bare Au. The real (black curve) and imaginary (red curve) parts of the dielectric permittivity for this case are shown in **Figure S1d**, together with the measured and fitted associated ellipsometrical angles Ψ (**Figure S1e**) and Δ (**Figure S1f**). A highly lossy ENZ transition around 400 nm is found (labeled “ENZ Au” in **Figure S1d**), determining the transition from a highly-lossy metallic spectral region to a highly lossy dielectric one. Such an effect highlights the capability of the random nano-structuration of the layer to dramatically change the optical behavior of a material passing, like in this case, from a metallic behavior to a dielectric one. The parameters of the Gaussian oscillators used to fit the dielectric permittivity of the nano-porous Au layer are reported in **Table S2**:

**Table 2:** Fitting parameters for the Gaussian oscillators used to fit the dielectric permittivity of the nano-porous Au samples.

|  | ***E_0_* (eV)** | ***γ_i_* (eV)** | ***A_i_*** |
| --- | --- | --- | --- |
| **Oscillator 1** | 1.67 | 3.29 | 14.17 |
| **Oscillator 2** | 2.09 | 0.58 | 2.79 |
| **Oscillator 3** | 2.89 | 0.57 | 7.164 |
| **Oscillator 4** | 3.23 | 0.39 | 6.21 |
| **Oscillator 5** | 3.69 | 1.62 | 8.30 |

**Supporting note #2: SNOM Analysis**

To experimentally support the numerical evaluation of the plasmonic near field calculated through COMSOL-based simulations in the main manuscript, we carried out Scanning Near-field Optical Microscopy (SNOM) experiments on the nano-porous Au films through an Alpha 300S SNOM apparatus by WITec used is transmission mode, exciting the structure with a continuous LASER source at 532 nm, focused directly into the hollow aluminum-coated SNOM tip (hollow diameter ~ 60 nm) through a 20x objective. Such a measurement provides both topographic and field information, allowing a fine overlap between topography and field maps. The topographic and near-field maps are shown in **Figure S2a,c** (10 μm × 10 μm) and **S2b,d** (20 μm × 20 μm) respectively showcasing good agreement with COMSOL based simulations, confirming that the field distribution is mainly determined by the excitation of plasmonic hotspots.


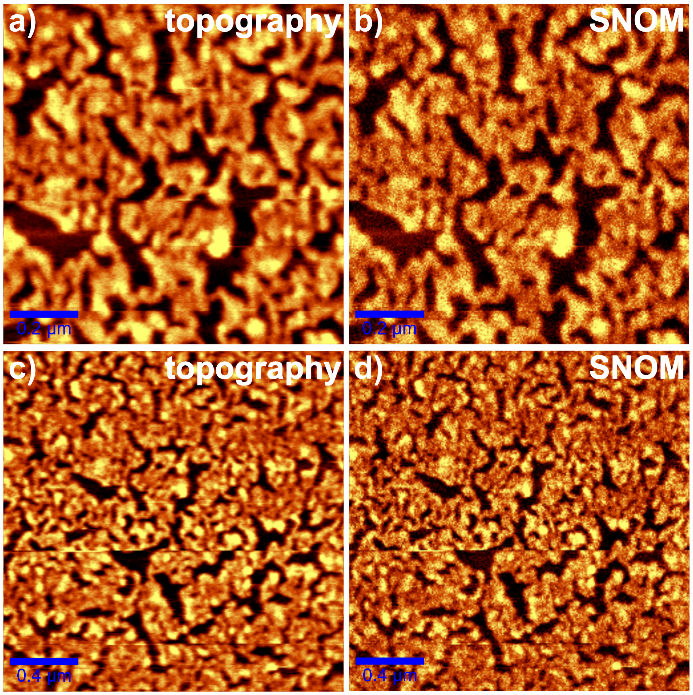


**Figure S2**: (a,c) Topographic and (b,d) SNOM analysis of (a,b) 10 μm × 10 μm and (c,d) 20 μm × 20 μm areas of the nano-porous Au sample.

**Supporting note #3: Pump-Probe measurements**

To experimentally access the electron mobility of the hybrid Ag-Au system and compare it with a standard pure Au NPM thin film, we performed pump probe measurements with sub-15 fs time resolution using a degenerate pump probe system^3^. As it can be inferred from Figure S3a, the interband transition around 600 nm is appearing within the spectral range accessible by our setup. The negative signal in transmission implies an excited state absorption due to the excitation of thermal electrons at the pump energy centered around 700 nm. The recovery time (red curve in Figure S3b) is 450 fs, indicating a similar dynamics than that observed in previous works on pure Au NPM thin films^4^ , thus confirming the high mobility and conducting behavior of the hybrid Ag-Au NPM system.


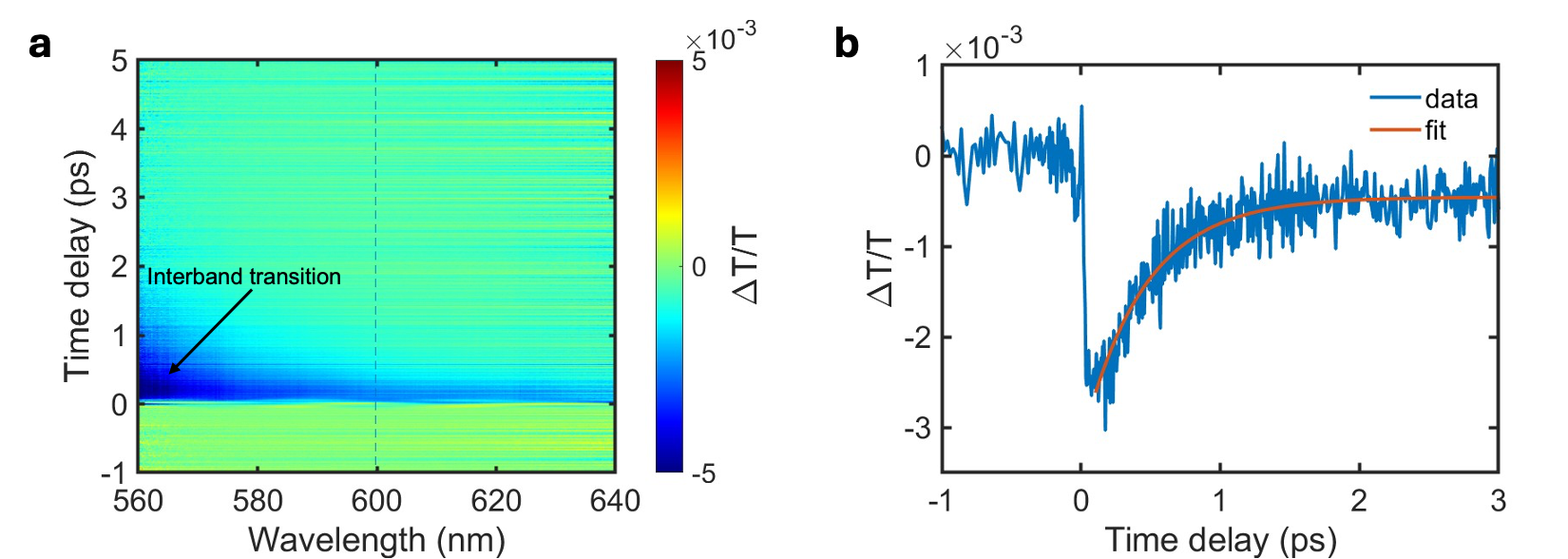


**Figure S3**: (a) 2D map of the differential transmission of the Au-Ag NPM thin film as function of the time delay between pump and probe and the wavelength of the probe light. (b) Differential transmission of the Au-Ag NPM thin film at 600 nm as a function of the time delay between pump and probe (dashed line in panel (a)). Decay time constant is 450 fs (red line fitting).

**Supporting note #4: Additional CL measurements**

| 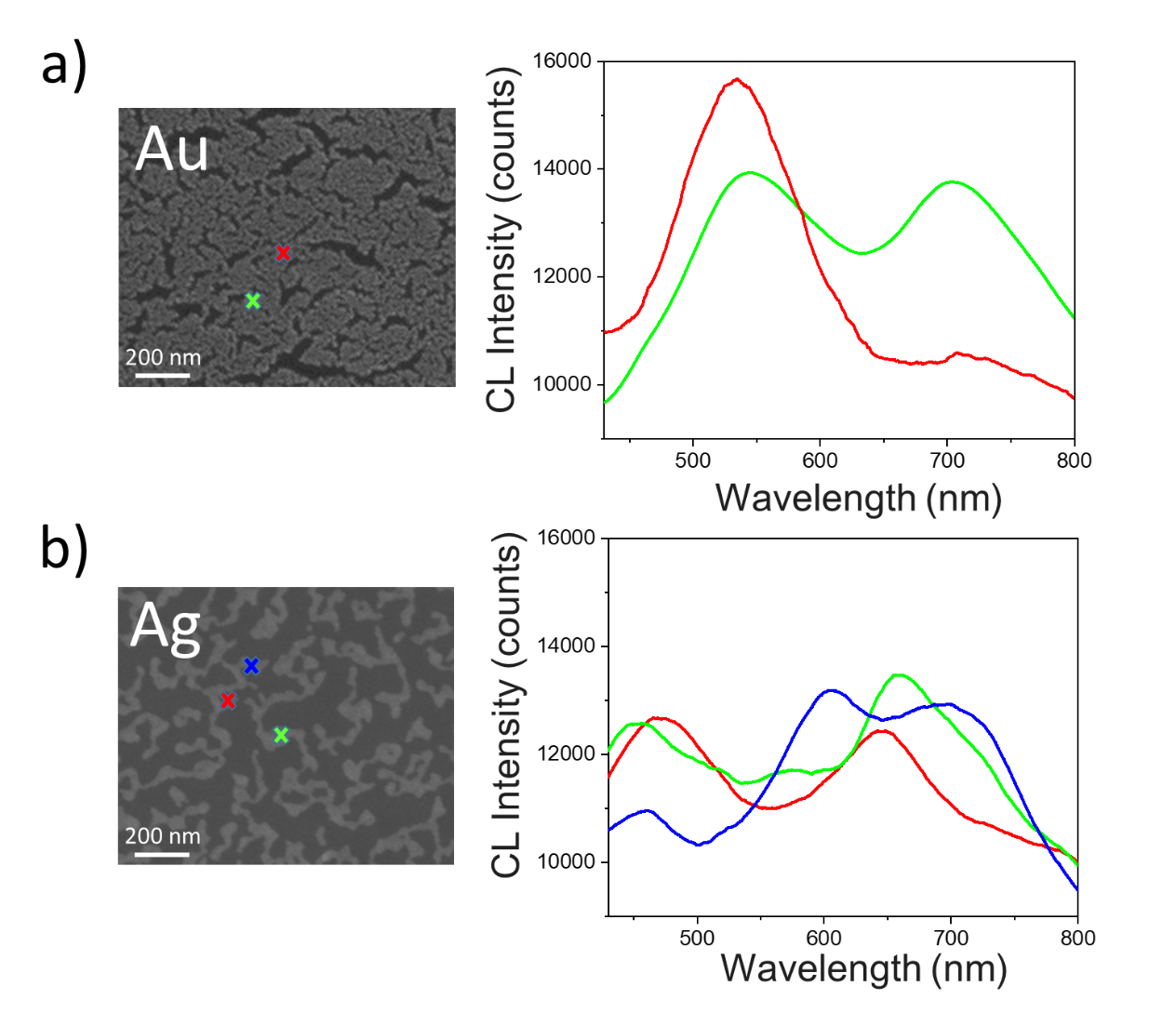 |
| --- |
| **Figure S4:** SEM image (500 nm × 500 nm) of the studied (a) Au and (b) Ag nanoporous networks, with related point CL spectra extracted from a few probe positions indicated by the corresponding colored crosses in the SEM image. |

We fabricated and characterized separate Ag and Au nanoporous networks (Figure S4a,b, left panels). In particular, we collected cathodoluminescence (CL) spectra from specific locations on both networks (Figure S4a,b, right panels). As observed, the Ag network^5^ exhibits a distinct emission peak at 460 nm, while the Au network does not. Interestingly, both networks display CL emissions within the 500-800 nm spectral range. Hence, the light spectra measured at various points on the Au-Ag network, marked by the yellow, green, blue, and red crosses in the SEM image of Figure 5a, are a result of combined emissions from both silver and gold layers within the network.

**Reference**

^1^ W. Newman, C.L. Cortes, J. Atkinson, S. Pramanik, R.G. DeCorby, and Z. Jacob, “Ferrell−Berreman Modes in Plasmonic Epsilon-near-Zero Media,” ACS Photonics **2**, 2–7 (2014).

^2^ R.A. Ferrell, “Predicted radiation of plasma oscillations in metal films,” Physical Review **111**(5), 1214–1222 (1958).

^3^ J. Kuttruff, D. Garoli, J. Allerbeck, R. Krahne, A.D. Luca, D. Brida, V. Caligiuri, and N. Maccaferri, “Ultrafast all-optical switching enabled by epsilon-near-zero-tailored absorption in metal-insulator nanocavities,” Communications Physics **3**(1), 1–7 (2020).

^4^ M. Ortolani, A. Mancini, A. Budweg, D. Garoli, D. Brida, and F. de Angelis, “Pump-probe spectroscopy study of ultrafast temperature dynamics in nanoporous gold,” Physical Review B **99**(3), 035435 (2019).

^5^ M. Scuderi, M. Esposito, F. Todisco, D. Simeone, I. Tarantini, L. De Marco, M. De Giorgi, G. Nicotra, L. Carbone, D. Sanvitto, A. Passaseo, G. Gigli, and M. Cuscunà, "Nanoscale Study of the Tarnishing Process in Electron Beam Lithography-Fabricated Silver Nanoparticles for Plasmonic Applications," J. Phys. Chem. C **120**, 24314−24323 (2016)
